# Estimating the cost and value of functional changes in communication ability following telepractice treatment for aphasia

**DOI:** 10.1371/journal.pone.0257462

**Published:** 2021-09-17

**Authors:** Molly Jacobs, Charles Ellis

**Affiliations:** 1 Department of Health Services Research, Management and Policy, University of Florida, Gainesville, Florida, United States of America; 2 Department of Speech, Language and Hearing Sciences, University of Florida, Gainesville, Florida, United States of America; The University of Hong Kong, HONG KONG

## Abstract

**Context:**

Aphasia is a post-stroke condition that can dramatically impact a person with aphasia’s (PWA) communication abilities. To date, few if any studies have considered the cost and cost-effectiveness of functional change in aphasia nor considered measures of patient’s value for aphasia treatment.

**Objective:**

To assess the cost, cost-effectiveness, and perceived value associated with improved functional communication in individuals receiving telerehabilitation treatment for aphasia.

**Design:**

Twenty PWA completed between 5 and 12 telehealth rehabilitation sessions of 45–60 minutes within a 6-week time frame using a Language-Oriented Treatment (LOT) designed to address a range of language issues among individuals with aphasia. National Outcomes Measures (NOMS) comprehension and verbal expression and the ASHA Quality of Communication Life (QCL) were completed prior to and at the completion of rehabilitation to obtain baseline and treatment measures.

**Results:**

Age, education, and race are significantly correlated with improvement in the NOMS verbal expression. African Americans (OR = 2.0917) are twice as likely as Whites to experience improvement after treatment. The likelihood of improvement also increases with each additional year of education (OR = 1.002) but decrease with age (OR = 0.9463). A total of 15 PWA showed improvement in NOMS comprehension and nine patients showed improvement in NOMS verbal expression. Improving patients attended between five and 12 treatment sessions. The average cost of improvement in NOMS comprehension was $1,152 per patient and NOMS verbal expression was $1,128 per patient with individual treatment costs varying between $540 and $1,296. However, on average, the monetary equivalent in patient’s improved QCL was between $1,790.39 to $3,912,54—far exceeding the financial cost of treatment.

**Conclusions:**

When measuring the functional improvement of patients with aphasia, patient’s quality of communication life received from treatment exceeded financial cost of services provided.

## Introduction

Aphasia is a post-stroke condition that can dramatically impact a stroke survivor’s listening comprehension, verbal expression, reading and writing [1]. In the US alone, 18% of all stroke survivors are discharged from hospital in with aphasia [2] and there are believed to be more than 2.5 million Americans are currently living with this condition [3]. Even very mild forms of aphasia can result in social isolation for the person with aphasia (PWA) and have been shown to reduce their ability to engage in society [4–6]. Many individuals with aphasia require long-term rehabilitation care to manage their aphasia resulting in greater overall costs of care for stroke survivors with aphasia when compared to those without aphasia [7,8]. This is a major concern in the US given the nation’s legislative efforts such as the Patient Protection and Affordable Care Act (PPACA) that was designed to control and subsequently reduce costs of care [9].

Recently, there has also been a greater emphasis placed on understanding not only the cost care for conditions like aphasia but also the cost-effectiveness of treatments designed to reduce the communication disability associated with aphasia and the associated benefits. For example, Palmer and colleagues [23], examined the cost-effectiveness of a computerized treatment for aphasia and found that the treatment yielded an incremental cost-effectiveness ratio (ICER) of $4,900, which indicated that the intervention was cost-effective [10]. Similarly, Wenke et al. found that computer-based and group treatment was 30% cheaper than standard service [11]. Finally, Jacobs and colleagues examined the cost-benefit of telepractice treatment for aphasia and found that each one-point reduction in impairment cost between approximately US $200 for those who improved and cost-benefit figures were influenced by aphasia type/severity with the lowest costs per reduction of impairment being observed by those with the most severe aphasia [12].

To date, few if any studies have considered the cost and cost-effectiveness of functional change in aphasia. Traditionally studies of aphasia utilize measures of impairment such as the Western Aphasia Battery-Revised (WAB-R) [13] which offer a “quotient” or index score of ability derived from subtests. However, quotient scores can be deceiving because a person with primarily comprehension deficits may achieve a very similar severity score to someone with primarily deficits of expression as a result of the method by which WAB-R scores are calculated and weighted. We do not argue against the importance and use of impairment scores such as the WAB-R yet measures of functional communication ability may be easier for patients/families to understand. At the same time a better understanding of communication performance can be translated into measures of value of the services received. Little is known about measure of value in the study of aphasia or any adult-onset neurogenic communication disorder. Understanding value is important in the study of rehabilitation outcomes for conditions like aphasia because value itself is derived from the perspective of the person receiving treatment (i.e PWA). According to Porter p. 4 [14], value represents the “health outcome per dollar of cost expended” and value represents what is most important to patients as it monetizes the services received [15].

The objective of this study was to examine the cost and cost-effectiveness of functional change in communication ability while also estimating the patient’s sustained value of services received through a telehealth platform. For this study we utilized the National Outcomes Measurement System (NOMS) [16] to measure change in functional communication and American Speech Language and Hearing Association Quality of Communication Life Scale (ASHA QCL) [17] to measure quality of communication outcomes. We utilized these measures to because they provide a distinct value for both functional improvement (NOMS) of the patient as well as the tangible post-treatment, enduring benefit to patients (ASHA QCL).

## Methods and procedures

This study was approved by the East Carolina University Institutional Review Board. Written consent of approval was provided to the authors. The study complies with all standards therein.

Study participants were 20 PWA recruited a part of a telerehabilitation study for aphasia. The clinical details of the study participants and information related to the aphasia telerehabilitation treatment approach were previously published [12]. In brief summary, study participants were recruited from the eastern region of North Carolina (NC). This area is known for high disease burden faced by residents with limited access to care. The aphasia rehabilitation treatment was delivered via a videoconferencing program that allowed real-time exchange of video and audio. This platform allowed the participants to see both the treatment stimuli and clinician simultaneously. Individuals with aphasia received the Language-Oriented Treatment (LOT) at a remote community-based site (local school or senior centre). Each participant completed between 5 and 12 telehealth rehabilitation sessions of 45–60 minutes within a 6-week time frame [18,19]. NOMS comprehension and verbal expression and the ASHA QCL were completed prior to and at the completion of rehabilitation to obtain baseline and treatment measures. For cost calculations, the cost of each session was based on 92507 CPT code (speech-language treatment) at a rate of $108.00.

### Main outcome measures

#### Functional communication

NOMS verbal and NOMS comprehension were utilized to assess pre- and post-treatment functional communication. NOMS are clinician reported measure of functional communication outcome [16]. NOMS utilizes a series of seven-point Likert assessment scales to obtain functional abilities and over multiple time points. A score of one indicates minimal or no ability whereas a score of seven indicates independence in the measured area (verbal expression and comprehension).

#### Quality of communication life

The ASHA-QCL was utilized to measure quality of communication life. The ASHA-QCL is a patient-reported measure of quality of communication that uses visual analog scales designed to measure quality of communication life from the perspective of adults with communication disorders [17]. The ASHA-QCL includes 17 statements designed to elicit the PWA’s rating of specific behaviors and skills and a final statement designed to measure their perception of overall QoL.

#### Aphasia costs

To calculate costs of aphasia treatment, the total billed cost of treatment was calculated as the total cost of all treatment sessions attended.

#### Value

Pre- and post-treatment ASHA Quality of Communication Life (QCL) was used to calculate the value of treatment and a monetary value was derived as the relative cost of care.

### Study cost and benefit framework

**Fig 1:**
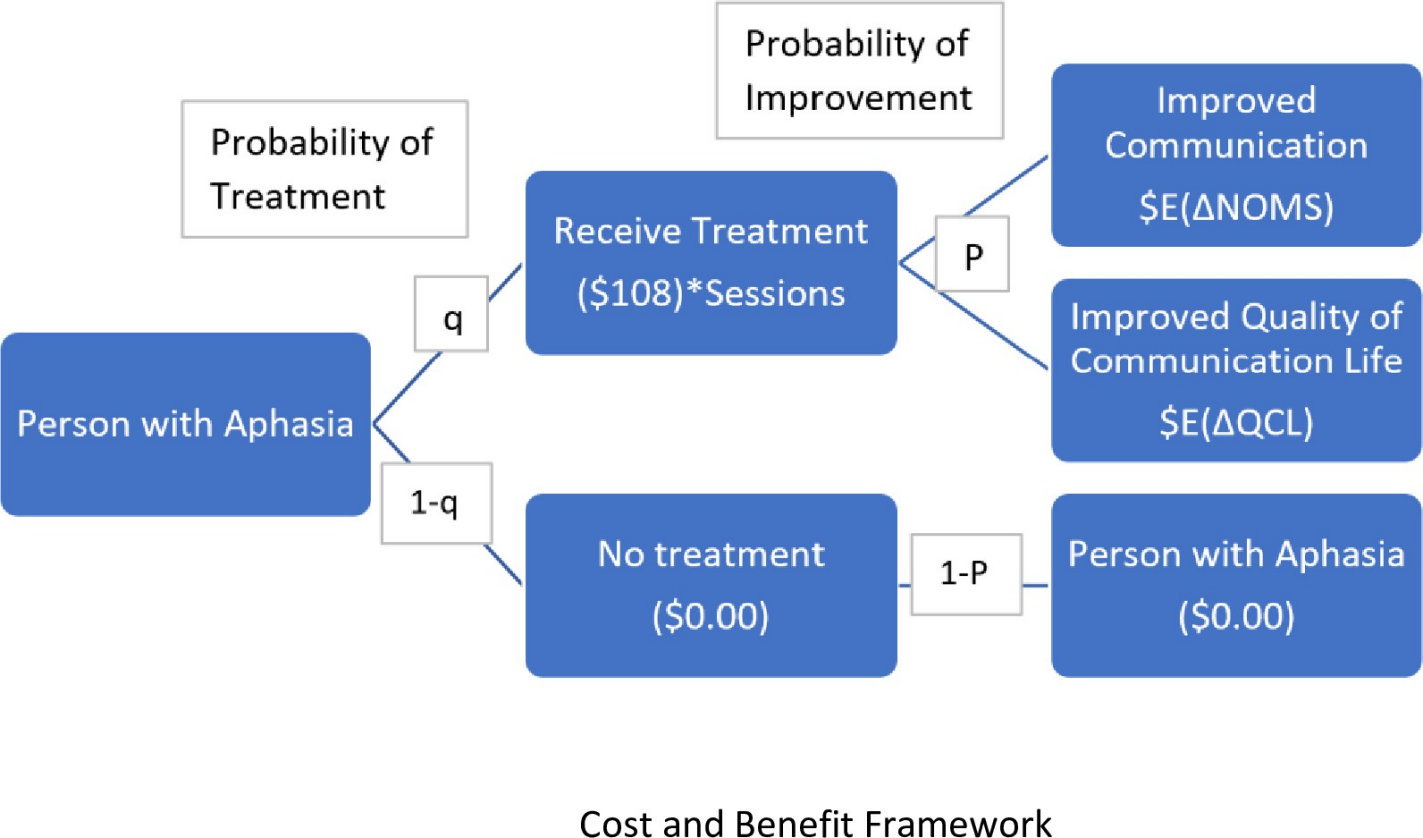
Cost and benefit framework.

See Fig 1 for the framework utilized to guide this study. The framework shows the relationship between receipt of treatment vs no treatment, cost treatment and sustained value of receiving treatment. The framework allows the intangible benefit of treatment to be translated to a monetary value.

### Statistical analysis approach

Excessive cost, sample attrition, neglect and noncompliance often result in small sample sizes in clinical trials. Therefore, several statistical methods have been presented to enable statistical inference with limited observations. One approach utilized in this study is Bayesian estimation. Rather than relying on large samples, Bayesian estimation allows the introduction of prior information in the form of probability distributions into the estimation procedure. This “prior” information is utilized to update information in the Bayesian algorithm along with information from the data itself. Due to their flexible implementation and interpretation, Bayesian methods have become an increasingly popular method of small sample estimation in clinical studies in the field of speech-language pathology and audiology. The quality of Bayesian estimation results are highly sensitive to the prior imposed; therefore, iterative model estimation was conducted to ensure the proper specification and distributional assumptions. One attractive feature of these models is that they allow for a variety of data structures, types of dependent variables, and distributional assumptions of the underlying mechanism.

### Calculating functional improvement

Estimation utilized improvement in NOMS verbal and NOMS comprehension score. Since clinicians score both NOMS verbal and comprehension on a 7-item Likert scale, individual improvements cannot be accurately compared using the nominal value of change. Therefore, a dichotomous dependent variable was created equaling one if the patient’s NOMS score improved with treatment, and zero otherwise. Binomial logistic regression tested the association between improvement in NOMS score and patient age, time post onset, education, and race. Given the variety of aphasia types among patients in this study, models were adjusted to allow for nesting, or clustering of different aphasia types within groups of patients who experienced NOMS improvement and those that did not. Therefore, the general aim of binomial logistic regression is to estimate the odds that a patient would experience a change in their NOMS score while taking the dependency of data into account—the fact that various aphasia types are nested within each group. Given that estimates from the regression coefficient (which represent the change in the log-odds of the predictor variable) are difficult to interpret, odds-ratio estimates are also provided.

To contextualize these results within the telerehabilitation framework, the likelihood of improvement is estimated for each patient based on their individual characteristics, type of aphasia, TPO, age, and race. This probability of improvement is used to calculate the expected value of improvement. In statistics, the expected value is calculated by multiplying the outcome by its likelihood of occurrence. In the context of this study, the expected value of improvement is the product of the cost of treatment and the probability of improvement. In other words, the cost of treatment is a weighted average of each patient’s treatment cost where the weights are the probability that the patient will improve with treatment. For those patients with a higher probability of improvement, the treatment has a higher monetary value than for those patients with a lower probability of improvement. Therefore, it is more valuable to those with the highest potential gain from improvement.

### Calculating costs

The average cost of improvement was calculated for both NOMS Verbal and NOMS Comprehension. Using an average billable fee of $108, the total cost of all treatment sessions is calculated for those individuals showing improvement. This figure is then averaged across respondents to calculate an average, per-person cost of improvement (See formula below).


p=Probabilityofexperiencingimprovementinfunctionalcommunication

NetCost=CostofTreatment*NumberofTreatmentSessions

E(Cost)=E(p*$108*#TreatmentSessions)+E((1-p)*$108*#TreatmentSesiions)

### Calculating value

To gauge the long-term value of improved communication on patients’ lives, a cost-benefit analysis was completed to calculate change in QCL following treatment as an incremental patient cost per unit QCL. The outcome of this analysis represents the value of the intervention to PWA (See formula below).


p=Probabilityofexperiencingimprovementinfunctionalcommunication

NetBenefit=ΔNOMS+ΔQCL

$$E(Benefit)=E(p*(\Delta NOMS+\Delta QCL))+E((1-p)*(NOMS_{initial}+QCL_{initial}))$$

## Results

Twenty individuals with aphasia completed the telerehabilitation study. Descriptive statistics (Table 1) showed that the sample was predominantly white with an average educational level of 14 years (post-high school). Participants ranged in age from 33 to 96 with a mean age of 61. Eight patients were diagnosed with Broca’s aphasia and nine patients had anomic aphasia. The remainder had either global (N = 1) or conduction aphasia (N = 2).

**Table 1:** Study sample characteristics.

	Full Sample	Anomic	Broca’s	Conduction	Global
Treatment Group Frequency					
N	20	9	8	2	1
African American	5	2	2	0	1
White	15	7	6	1	0
NOMS Verbal Improvement	9	4	2	2	1
NOMS Comp Improvement	15	6	6	2	1
Treatment Group Mean (Standard Deviation)					
Age	60.9 (15.2)	65.6 (13.1)	56 (13.7)	59 (17.0)	35 (-)
TPO	40.4 (64.1)	24.4 (21.4)	64.1 (97.3)	11 (7.1)	16 (-)
Education	14.6 (2.3)	14.9 (3.0)	14.1 (1.6)	14 (2.8)	16 (-)
NOMS Verbal Initial	4.5 (1.7)	5.9 (0.6)	3.5 (1.4)	3.5 (0.7)	1 (-)
NOMS Verbal Final	4.9 (1.7)	6.3 (0.7)	3.8 (1.2)	4.5 (0.7)	2 (-)
NOMS Comp Initial	5.05 (1.4)	5.9 (0.9)	4.8 (0.7)	4.5 (0.7)	1 (-)
NOMS Comp Final	6.05 (1.0)	6.7 (0.7)	5.6 (0.9)	6 (0.0)	4 (-)

### Functional treatment outcomes

Most patients completed all scheduled treatment sessions (13 patients attended all offered sessions). However, number of sessions attended did not directly correlate with improvement. For example, one patient who attended all sessions showed no improvement on either NOMS verbal or comprehension, while a patient who attended only eight sessions improved on both outcomes.

Less than half of patients (N = 9) experienced improvement in NOMS Verbal, while 75 percent (N = 15) experienced improvement in NOMS Comprehension. All patients with global and conduction aphasia improved in both NOMS Comprehension and NOMS Verbal. However, only 25 and 44 percent of patients with Broca’s and anomic aphasia improved in NOMS verbal. A higher proportion, 75 and 67 percent, of individuals with Broca’s and anomic aphasia improved on NOMS comprehension after treatment. Table 2 shows improvement by aphasia type for both scales. Those patients with highest level of impairment (lowest initial scores) improved the most over the six weeks.

**Table 2:** Bayesian binomial logistic regression: Factors related to NOMS verbal improvement.

	Estimate	Std Dev	Effect Size	Rhat	95% Posterior Interval		Odds Ratio
Intercept	**4.143**	6.440	1986	1.002	-6.235	14.944	
Age	**-0.055**	0.051	2674	1.000	-0.143	0.025	0.946276
Months Post Onset	-0.028	0.018	3165	1.000	-0.061	-0.001	0.97269
Education	**0.002**	0.299	2568	1.002	-0.483	0.495	1.001866
African American	**0.738**	1.600	2246	1.001	-1.901	3.378	2.091714
Intercept(anomic)	**0.122**	0.954	2047	1.001	-1.412	1.733	1.129753
Intercept(broca)	**-1.020**	1.239	1664	1.002	-3.474	0.338	0.360688
Intercept(conduction)	**0.795**	1.437	3026	1.001	-0.759	3.647	2.213615
Intercept(global)	0.283	1.459	3369	1.000	-1.655	2.738	1.326895

Depend Variable: 1 = Improvement in NOMS Verbal, 0 = No improvement **Indicates** statistically significant.

We note that mean values are not adequate to determine the statistical relevance of these observations given that the measurements were completed on a Likert scale and a range of sociodemographic characteristics contribute to clinical outcomes. Additionally, subtype cohorts are relatively small for several groups. Therefore, implementation of data augmentation for Bayesian regression evaluated the impact of race, age, education, and type of aphasia on the likelihood of experiencing improvement in NOMS verbal and NOMS comprehension. In Bayesian analysis, a parameter is summarized by an entire distribution of values known as the posterior distribution. This distribution is approximated using the observed data and applies Markov chain Monte Carlo (MCMC) methods. Bayesian inference uses the posterior distribution to form various summaries for the model parameters, including point estimates, standard deviations and posterior interval estimates known as credible intervals. Estimation results are listed in Tables 2 and 3. In discrete dependent variable models, coefficients are interpreted as the difference between the log of expected counts corresponding to a one unit change in the predictor variable. To provide a more intuitive, odds ratio estimates are also listed.

**Table 3:** Bayesian binomial logistic regression: Factors related to NOMS comprehension improvement.

	Estimate	Std Dev	Effect Size	Rhat	95% Posterior Interval		Odds Ratio
Intercept	-15.113	8.557	2107	0.999	-29.719	-1.983	
Age	0.146	0.068	2155	1.000	0.042	0.265	1.157206
Months Post Onset	**-0.004**	0.012	2828	1.002	-0.022	0.017	0.996202
Education	**0.505**	0.401	2681	0.999	-0.121	1.180	1.657424
African American	**0.814**	2.106	2377	0.999	0.547	7.455	2.256798
Intercept(anomic)	**-0.886**	1.191	1956	1.000	-3.221	0.449	0.412442
Intercept(broca)	**0.417**	1.048	2101	1.003	-0.994	2.426	1.517683
Intercept(conduction)	**0.924**	1.545	2293	1.000	-0.625	3.886	2.519184
Intercept(global)	0.425	1.433	2875	1.000	-1.394	3.146	1.529631

Depend Variable: 1 = Improvement in NOMS Comprehension, 0 = No improvement **Indicates** statistically significant.

Age, education, and race are significantly correlated with improvement in the NOMS verbal. African Americans (OR = 2.0917) are twice as likely as Whites to experience improvement after treatment. The likelihood of improvement also increases with each additional year of education (OR = 1.002) but decrease with age (OR = 0.9463). Improvement in NOMS comprehension was associated with months post onset, education, and race. PWA were less likely to improve as their months post onset increased (OR = 0.9963), but over 50 percent more likely to experience improvement from treatment as their years of education increased (OR = 1.6574). African Americans are twice as likely to improve compared to Whites (OR = 2.2567). Regarding aphasia type, the type of aphasia was associated with odds of improvement for both NOMS comprehension and verbal expression. Individuals with more severe forms of aphasia, such as global, had a larger margin for improvement than those with anomic or Broca’s.

### Cost outcomes

Table 4 shows the number of patients improving in both NOMS comprehension and Verbal, the number of visits each patient attended, and the average cost of improvement for each patient who improved. A total of 15 patients showed improvement in NOMS Comprehension and nine patients showed improvement in NOMS Verbal. Improving patients attended between six and 12 treatment sessions. The average cost of improvement in NOMS comprehension was $1,152 per patient and NOMS Verbal was $1,128 per patient with individual treatment costs varying between $540 and $1,296.

**Table 4:** Average cost of improvement.

	Patients	Visits	Cost	Total	Per Patient
NOMS Comprehension	15	5 to 12	$540 to $1296	$17,280	$1,152
NOMS Verbal	9	6 to 12	$648 to $1296	$10,152	$1,128

### Value outcomes

Finally, Table 5 contains the cost-benefit analysis designed to examine the value of aphasia treatment. Using the probability of improvement for each patient, the expected cost of improvement was calculated based on the treatment cost and likelihood of improvement. Since the value of improvement was assumed to be the improvement in QCL, the value of improvement is the monetary value for each unit QCL. These values are summarized by aphasia type. For patients with global or conduction aphasia, the value of improvement far exceeds the cost. However, for patients with less impairment, the range of values varies more widely—a result of the wider variation in the probability of improvement among these patients.

**Table 5:** Simulated sample type, probability of improvement, cost of improvement and value of improvement in QCL.

	Total Cost	Probability of Improvement		Expected Cost			Value of Improvement	
Type	Total Treatment Cost	P(Verbal Improvement)	P(Comp Improvement)	E(Cost Verbal Improvement)	E(Cost of Comp Improvement)	QCL Improvement	E(Value of QCL Improvement)^VERBAL^	E(Value of QCL Improvement)^Comprehension^
Anomic	1188	72%	95%	$ 850.61	$ 1,127.06	1.12	$ 759.47	$ 1,006.30
Anomic	648	77%	32%	$ 501.68	$ 207.17	1.15	$ 436.24	$ 180.14
Anomic	864	36%	78%	$ 306.72	$ 670.64	0.59	$ 519.86	$ 1,136.67
Anomic	540	42%	94%	$ 224.80	$ 506.90	1.4	$ 160.57	$ 362.07
Anomic	972	42%	59%	$ 411.16	$ 571.24	0.34	$ 1,209.28	$ 1,680.13
Anomic	1080	28%	93%	$ 299.16	$ 1,006.88	1.07	$ 279.59	$ 941.01
Anomic	1296	54%	90%	$ 695.56	$ 1,160.96	1.06	$ 656.19	$ 1,095.24
Anomic	648	51%	91%	$ 333.07	$ 587.93	1.13	$ 294.75	$ 520.29
Anomic	1296	27%	65%	$ 350.83	$ 840.46	0.23	$ 1,525.34	$ 3,654.16
Broca’s	1296	70%	46%	$ 906.81	$ 598.36	0.2	$ 4,534.06	$ 2,991.82
Broca’s	1296	3%	70%	$ 33.37	$ 909.79	0.34	$ 98.15	$ 2,675.86
Broca’s	1296	14%	73%	$ 180.14	$ 939.60	0.54	$ 333.60	$ 1,740.00
Broca’s	1296	17%	71%	$ 219.67	$ 923.40	0.25	$ 878.69	$ 3,693.60
Broca’s	1296	32%	89%	$ 413.68	$ 1,149.55	0.03	$ 13,789.44	$ 38,318.40
Conduction	1296	81%	61%	$ 1,050.67	$ 786.41	0.56	$ 1,876.19	$ 1,404.31
Global	1296	85%	79%	$ 1,100.56	$ 1,020.60	0.85	$ 1,294.78	$ 1,200.71
				E(Cost)	E(Value)			
			Anomic	$207.17-$1,160.96	$180.14-$3,654.16			
			Broca’s	$219.67-$1,149.55	$333.60-$38,318.40			
			Conduction	$786.41-$1,050.67	$1,404.31-$1,876.19			
			Global	$1,100.56-$1,020.60	$1,200.71-$1,294.78			

## Discussion

To date, few studies have examined the cost and cost-effectiveness of functional improvement in aphasia. Understanding functional improvement in aphasia and other post-stroke disorders is important because functional outcomes are the most meaningful patient outcomes [20]. Interestingly, in the field of aphasia, studies primarily focus upon impairment level measures which have less meaning for PWA and their families. Functional communication outcomes in aphasia are critically important for demonstrating value and effectiveness of services [21]. According to Mullen, over 70% of PWA who receive speech-language pathology (SLP) services demonstrate significant functional improvement [21]. Yet, despite the utilization of functional measures for quality measurement and reimbursement, few studies report functional aphasia outcomes [22]. There remains a lack of consensus how to specifically measure functional communication among PWA [22].

The study results reported here indicated that PWA were more likely to demonstrate functional improvement in the area of auditory comprehension relative to verbal expression. These findings are important because when families/partners of aphasia rate the communication effectiveness of PWA, the reported rating primarily reflect expression ability even though success communication also requires listening comprehension [23]. The observations of greater comprehension improvements likely reflect differential recovery patterns within a generally small sample of PWA as well as the type and severity of aphasia at the onset of the study. We do not draw a clear conclusion that comprehension skills are more likely to recover than expression. In particular, recovery of functional communication depends upon their language impairment as well as disruption in cognitive processes as well as verbal and non-verbal abilities [24]. Regardless, the long-term improvements translate into improved quality of life and greater engagement and participation in society. Further, studies of long-term recovery have yielded mixed results with some suggesting greater comprehension recovery long term and other greater verbal expression recovery [25]. Finally, it is also notable that improvements were influenced by a range of clinical (aphasia type/severity) and sociodemographic (age, education, race) characteristics. Interestingly, race as a factor influencing functional improvements have been previously reported. African Americans with aphasia exhibited less improvement than non-Hispanic Whites (Whites) with aphasia in verbal expression, auditory comprehension, reading, and writing even when controlling for treatment severity and aphasia severity [21].

Although an emerging literature exists related to cost and cost-effectiveness of treatments for aphasia using measures of impairment [8,10–13], little is known about the cost and cost-effectiveness of improving functional communication in aphasia. As a comparison, our previous work showed that on average a one-point improvement in impairment as measured by the WAB-R AQ costs approximately $200 [12]. In contrast, a one level change in functional communication cost approximately $1100 for improvements in verbal expression or auditory comprehension. Because the literature is limited related to cost of treatments to achieve functional outcomes for aphasia, we have no data for general comparisons. Butzer and colleagues argue that the true rehabilitative costs of care are rarely measured and in order to facilitate cost-effective and value-based care, cost should be measured relative to the outcomes achieved [27]. We believe this work is a critical first step to better understand costs, cost-effectiveness and value of aphasia treatments.

In consideration of our concerns about comparative data for further evaluation of the cost findings reported here, we elected to complete a cost-benefit analysis designed to calculate change in QCL following treatment as an utilize incremental patient cost per unit of a quality of communication life measure to measure value from the PWA’s perspective. Measures of value of this kind are not new and offer unique and broader insights into treatments for conditions like aphasia. For example, Pauker & Kassirer argued that “expected value” can be calculated by obtaining a probability or measure of “utility” or “value” for the outcome. A recent call to address issues of value emerged from the State of the Science (SOS) Symposium on “The Value of Rehabilitation Interventions” which emphasized the need to utilize the current best evidence to demonstrate value of rehabilitation interventions [26]. It has become clear that a greater emphasis must be placed on identifying evidence-based treatments that offer the greater value to ensure that low value treatments are not overutilized or are the default practices [27].

For this study we utilized measures of quality of communication life to calculate the anticipated value of a change in functional communication (verbal expression or auditory comprehension). In addition, this study included measures of costs which are required to accurately estimate value [28]. Consequently, the study showed that value of treatment is higher for individuals with the most severe aphasias although value varied significantly across PWA. We acknowledge that relatively few studies have measured the cost of rehabilitative care, therefore, little precedence exists. The framework utilized herein provides what the authors feel is an accurate and logical methodology for evaluating this data and associated cost framework. Furthermore, these findings do agree with other treatment valuation studies that show greater improvements and lower costs per unit of aphasia impairment among individuals with the most severe aphasias [12].

### Study limitations

While the study reported here presented interesting results, a range of limitations should be considered. First, all outcome measures were derived using telepractice treatment for aphasia. Attempts to directly translate the reported findings to more traditional face-to-face approaches, alternative forms of delivery, or types of aphasia not included in this study should consider the contextual implications of these findings. Second, NOMS are clinician reported measures of functional communication. Some believe the that best measures of functional communication emerge from the perspective of the individuals with the communication disorder, rather than the treatment provider [24]. Consequently, differences in how PWA and clinicians report functional communication ability should be considered. Third, because the findings are based on measures of functional communication, they cannot be compared directly to studies reporting measures of impairment. Fourth, analysis of the NOMS outcomes were operationally defined as binary indicators of functional change (change vs no change) rather than magnitude of change which is frequently reported in studies of aphasia. Fifth, the study was based on a sample size of 20 PWA thus subsequent generalizations should be made with caution. Sixth, the study included a heterogenous group of PWA who differed significantly in their time post-onset and age.

## Conclusions

As telepractice treatments for aphasia are becoming more widely used, information related to the cost of care and cost-effectiveness of treatments provided is needed. Similarly, more information is needed to enhance the understanding or the relationship between impairment and functional communication measures utilized in aphasia treatment and quantify these relationships. Greater information about functional change following aphasia treatment can assist clients in the value of treatments offered and from their perspective. More importantly, measures of functional communication in aphasia can offer critical information about the cost of aphasia care required to demonstration functional improvements while also predicting the value of the treatment provided.
